# Enhancement of Activity of Thermophilic Inorganic Pyrophosphatase Ton1914 via Site-Directed Mutagenesis

**DOI:** 10.3390/biom15101395

**Published:** 2025-09-30

**Authors:** Siyao Liu, Xinrui Yang, Renjun Gao, Guiqiu Xie

**Affiliations:** 1 School of Pharmaceutical Sciences, Jilin University, Changchun 130021, China; siyao22@mails.jlu.edu.cn (S.L.); xryang23@mails.jlu.edu.cn (X.Y.); 2 Key Laboratory for Molecular Enzymology and Engineering of Ministry of Education, School of Life Sciences, Jilin University, Changchun 130021, China; gaorj@jlu.edu.cn

**Keywords:** inorganic pyrophosphorylase, thermophilic enzyme, site-directed mutagenesis, PCR enhancer, UDP-Gal synthesis

## Abstract

Inorganic pyrophosphatase (PPase) is an enzyme that catalyzes the hydrolysis of pyrophosphate (PPi) into two phosphates. Ton1914, a thermophilic inorganic pyrophosphatase derived from *Thermococcus onnurineus* NA1, has good thermal stability and an extremely high optimum temperature and has been shown to reduce pyrophosphate inhibition. In this study, eight sites were selected based on sequence alignment and software calculations, and multiple single mutants were successfully constructed. After saturation and superposition mutations, six superior mutants were obtained. The enzyme activities of E97Y, D101K and L42F were increased 2.57-, 2.47- and 2.15-fold, respectively, while those of L42F/E97Y, L42F/D101K and E97Y/D101K were increased 2.60-, 2.63- and 1.88-fold, respectively, relative to the wild-type enzyme. Compared to Ton1914, all mutants more effectively increased PCR product quantity, reduced the number of qPCR cycles required to reach the threshold, and improved the efficiency of gene amplification. In the UDP-Galactose (UDP-Gal) synthesis reaction, the addition of mutants could further improve yield. When Ton1914 and mutants with the same activity were added, the yield of UDP-Gal was almost identical, effectively reducing the dosage of pyrophosphatase. Overall, the mutants showed greater prospects for industrial application.

## 1. Introduction

Inorganic pyrophosphates (PPi) are common by-products of metabolic processes, participating in the biosynthesis of biological polymers, such as DNA, RNA, proteins, peptidoglycan, lipids (like cholesterol), cellulose and starches [1], and in post-translational modifications of proteins, including adenosylation, uridinization and ubiquitination [2]. PPi consists of two inorganic phosphate molecules linked by a hydrolyzable ester bond, primarily produced through the hydrolysis of phosphodiester bonds in triphosphorylated nucleotides (such as ATP or UTP), making it a metabolic by-product of many intracellular biochemical reactions and extracellular signaling cascades [3]. Breaking down PPi is an exothermic process in which in vitro biocatalytic models and real-world industrial production approaches often face the challenge of pyrophosphate accumulation. Therefore, the reactions catalyzed by inorganic pyrophosphatases can be coupled with processes that are unfavorable for biological transformation, promoting thermodynamic equilibrium toward biosynthesis [4]. Thermophilic inorganic pyrophosphatases originate from thermophilic bacteria that exhibit exceptional thermal stability. Owing to their beneficial functions, many inorganic pyrophosphatases expressed in such hyperthermophilic bacteria have been utilized by researchers [5,6,7,8,9,10].

PCR has served as a fundamental technique in the development of molecular biology. As a convenient and fast tool, it has been crucial in processes such as nucleic acid amplification and molecular modification [11]. PCR also has a wide range of analytical applications in the food [12], environmental [13], medical [14] and forensic fields [15]. It enables the continuous release of pyrophosphate by adding deoxyribonucleoside monophosphate from deoxyribonucleoside triphosphate during the primer extension process. Pyrophosphate can be hydrolyzed by PPase to two molecules of orthophosphate, which can kinetically reduce the inhibition of DNA elongation [16]. Therefore, the use of heat-stabilized PPase in PCR systems can increase the efficiency of product formation [17]. Ton1914 has been shown to contribute to the amplification of real-time fluorescence-based quantitative PCR and the degradation of by-products to reduce inhibition and increase the reaction rate and product quantity [18]. In nucleic acid research, thermophilic pyrophosphatase also plays an indispensable role in in vitro transcription [19,20] and gene sequencing [21], among other applications.

UDP-Gal is a widely used nucleotide sugar and key sugar donor in the production of galactosides, making it an essential component in pharmaceutical manufacturing. Light and thermal instability make it difficult to purify [22], leading to high costs and compromising its use as a raw material for galactoside synthesis [23]. To solve this problem, Li developed an efficient and economical one-pot, heat-resistant three-enzyme system for the large-scale synthesis of UDP-Gal [24]. In this system, Ton1914 plays a key role in reducing the inhibition of pyrophosphate and continuously promotes the reversible reaction catalyzed by UDP-glucose pyrophosphorylase in the direction of UDP-Gal generation, considerably impacting the yield of UDP-Gal.

*Thermococcus onnurineus* NA1 is a hyperthermophilic archaeon found in deep-sea hydrothermal vents. Three inorganic pyrophosphatases have been identified in *Thermococcus onnurineus* NA1, among which Ton1914 has been studied in our lab [18,25,26]. Previous studies characterized it and preliminarily explored its role in enhancing PCR and promoting UDP-sugar synthesis [18]. Ton1914 was found to improve the efficiency of polymerase chain reaction (PCR) and UDP-sugar synthesis by hydrolyzing PPi to Pi. In this study, site-directed mutagenesis was used to modify Ton1914 and obtain mutants with higher activity and greater stability than the wild-type enzyme. These mutants provide better options for enhancing PCR, qPCR and UDP-Gal synthesis owing to their increased ability to reduce pyrophosphate inhibition, further improving the catalytic performance and utilization value of Ton1914.

## 2. Materials and Methods

### 2.1. Chemicals and Reagents

A Fast Pfu DNA polymerase Kit, Quick Cut DpnI and T4 DNA Ligase were purchased from TransGen Biotech (Beijing, China). TB Green^®^ Premix Ex TaqTM was purchased from TaKaRa (Beijing, China). A Tianprep Mini Plasmid Kit and Universal DNA Purification Kit were purchased from Tiangen Biotechnology Company (Beijing, China). Isopropyl-β-d-thiogalactopyranoside (IPTG) was purchased from solarbio (Beijing, China), while galactose and UDP-Gal were purchased from Yuanye Biotechnology Co., LTD. (Shanghai, China). A Supersil AQ-C18 HPLC column was purchased from Eilit Analytical Instruments Co., LTD. (Dalian, China). Finally, chromatographically pure triethylamine, acetonitrile and methanol were purchased from Oceanpak Alexative Chemical Ltd. (Gothenburg, Sweden).

### 2.2. Strains and Plasmids

The strain *Thermococcus onnurineus* NA1 was purchased from JCM (Japan Collection of Microorganisms), while *Escherichia coli* BL21(DE3) was purchased from TransGen Biotech (Beijing, China). The plasmid pET28a (+) was obtained from our laboratory’s collection. Bacteria were cultured in Luria–Bertani (LB) medium at 37 °C.

### 2.3. Structure and Sequence Analysis of Ton1914

Discovery Studio 2022 (v21.1.0.20298, BIOVIA Discovery Studio, San Diego, CA, USA) was used to analyze the results of molecular docking. First, ChemDraw (v20.0) was used to draw pyrophosphate and optimize its structure, and then the protein structure was preprocessed using the Prepare Protein program of Discovery Studio, water molecules were removed and charge balanced, and CHARMm position was attached to the treated protein receptor and substrate ligand. The Pose Cluster Radius was defined as 0.5, and the best molecular docking result was selected according to the molecular orientation between the substrate and the active center. Consurf (https://consurf.tau.ac.il/consurf_index.php, accessed on 6 December 2022) was used to predict the site conservation and exposure of proteins, and Multalin (http://multalin.toulouse.inra.fr/multalin/, accessed on 3 January 2023) was used to align the amino acid sequence of Ton1914 with those of other inorganic pyrophosphorylases. Sequence analysis and mutation site selection were performed based on multiple-alignment results and HotSpot Wizard (http://loschmidt.chemi.muni.cz/hotspotwizard, accessed on 10 January 2023) calculations.

### 2.4. Construction of Ton1914 Mutants

The recombinant plasmid pET28a-Ton1914 was constructed previously. It was used as a template in Primer (v5.0, Premier BioSoft, Palo Alto, CA, USA) to design primers for the 15 selected mutants for whole-plasmid PCR. After the whole-plasmid PCR, the product yield was verified by means of agarose gel electrophoresis, and Dpn I was used to digest the pET28a-Ton1914 template gene. Subsequently, the agarose gel recovery kit was used to recover the digestion products. The mutant plasmid was then transformed into competent *Escherichia coli* BL21(DE3) cells using heat shock. The cells were then spread on a solid medium containing kanamycin and cultured for the subsequent selection of single-clone colonies.

### 2.5. Overexpression and Purification of Ton1914 Mutants

The recombinant plasmids of the pET28a-Ton1914 mutants were transformed into *E. coli* BL21 (DE3) strains. *E. coli* BL21 (DE3) carrying the recombinant plasmid was grown overnight at 37 °C and 180 rpm in Luria–Bertani (LB) medium (6 mL) with kanamycin, transferred to fresh LB medium (200 mL) containing kanamycin and incubated at 37 °C for an additional 8 h, with the addition of a final concentration of 0.5 mM IPTG when the OD_600_ of the culture reached 0.8. The recombinant protein was then induced at 25 °C and 120 rpm for 12 h. Next, *E. coli* BL21 (DE3) cells were collected by centrifugation. The pellet was resuspended in an appropriate amount of 50 mM Tris-HCl (pH 9.0), and the bacterial cells were disrupted using ultrasonic dismemberment. After centrifugation at 13,980× *g* for 15 min, the crude enzyme solution was placed in a water bath at 80 °C for 10 min to denature the host cell proteins, followed by centrifugation once more to remove turbid impurities. The supernatant then was concentrated using an ultrafiltration tube to obtain purified protein, and the protein concentration was determined by BCA protein quantification. Finally, aliquots of the purified enzymes were stored at −60 °C.

### 2.6. Enzyme Assay

The amount of PPase required to produce 1 μmol of phosphate in one minute of pyrophosphate synthesis was defined as 1 U. Under acidic conditions, the complexation of phosphate, ammonium molybdate and ferrous ions formed a blue complex. Absorbance at 660 nm was measured to assess enzyme activity. The reactions involving Ton1914 and its mutants occurred in a solution of 1 mM sodium pyrophosphate, 2 mM Mg^2+^, 50 mM Tris-HCl and an appropriate amount of enzyme, with a total volume of 1 mL. The reaction system was incubated at 80 °C for 2 min and then mixed with 100 μL of a 100 mM citric acid solution and placed on ice for two minutes to stop the reaction. Then, 500 μL of the enzyme-catalyzed reaction mixture was added to 500 μL of Taussky–Shorr reagent, mixed and allowed to react at room temperature for 10 min. Next, 900 μL of the mixed solution was removed and measured for absorbance at 660 nm. For each experiment, three parallel controls were included to ensure measurement accuracy. Taussky–Shorr reagent was composed of 10% ammonium molybdate solution dissolved in 5 M H_2_SO_4_ and 0.05 g/mL Fe_2_SO_4_ in 10% ammonium molybdate solution.

### 2.7. Characterization of Enzymatic Properties

Determination of the optimal reaction temperature: An appropriate amount of Ton1914 and each mutant was allowed to react at 30–100 °C. The optimum temperature was determined according to the enzyme activity levels at different temperatures.

Determination of the optimal reaction pH: A pH range of 6–11 was selected, and the buffer solutions used were a 50 mM Tris-HCl buffer solution (pH 6.0–9.0) and 50 mM Gly-NaOH buffer solution (pH 9.0–11.0). Enzyme activity was determined at 90 °C.

Temperature stability test: Ton1914 and its mutants were incubated at 80 °C for 0–10 h and at 90 °C for 0–6 h. After incubation at these high temperatures, the enzyme liquid was cooled, and the activity of Ton1914 and each mutant was measured.

Thermal stability: The Tris-HCl buffer solution, serving as a control, was placed in a differential scanning calorimeter (DSC) with Ton1914 and each mutant at a concentration of 0.1 mg/mL. After heating at a set rate, a DSC thermal analysis diagram was obtained, with temperature as the abscissa and the heat required for there to be no temperature difference between the sample and reference product as the ordinate. Based on this curve, Tm (melting temperature) was analyzed.

Determination of kinetic parameters: The concentration of pyrophosphate was set to 0.05–1.0 mM according to the gradient. The enzyme activity (U/mg) at different substrate concentrations was obtained at 90 °C and pH 9, after which the *K_m_* (mM) values for each mutant were calculated using GraphPad Prism 7.0. Then, *k_cat_* (s^−1^) and *k_cat_*/*K_m_* (mM^−1^·s^−1^) were calculated according to the formula *k_cat_* = *Vmax*/[E].

### 2.8. Molecular Dynamic Simulation

GROMACS (v2022.3) was used for molecular dynamics simulations of Ton1914 and its mutants. Firstly, the protein molecules were attached with an OPLS-AA/L all-atom force field, and the SPC/E water model was used for solvation treatment. Then the protein molecules were placed at the center of the cube, and the minimum distance from the boundary was specified as 1.0 nm. After the solvent molecules were added to the model, thirteen solvent molecules were replaced with negative charges in the sodium ion equilibrium system. The F_max_ tolerance is set to 1000 kJ/(mol·nm). Energy minimization of the protein structures was performed using the steepest descent integrator (500 steps) and a conjugate gradient algorithm (500 steps). Furthermore, slow heating from 0 to 300 K of each system were performed, and those were equilibrated under a constant volume (NVT) ensemble and a constant pressure (NPT) ensemble at 300 K for 50 ps, followed by MD simulation for 100 ns at 300 K. Trajectory analyses included root mean square deviation (RMSD) and root mean square fluctuation (RMSF).

### 2.9. Enhancement of the Efficiency of PCR Reaction

The Ton1914 mutants were used to improve PCR efficiency in experiments containing 1 μL of multiple template genes, 0.5 μL of an upstream primer, 0.5 μL of a downstream primer, 1 μL of Mg^2+^, 10 μL of the 5× Fast Pfu Buffer, 4 μL of a dNTP solution, 29 μL of sterile water, 0.5 μL of Fast Pfu DNA polymerase and 3.5 μL of Ton1914 and its mutant enzyme solution, amounting to 50 μL in the PCR system. The number of the amplification cycles was set to twenty. The PCR results were analyzed by means of DNA agarose gel electrophoresis. The PCR product bands were quantified using ImageJ (v1.8.0.345) for grayscale analysis. The band intensity of the group supplemented with Ton1914 was used as the reference. For each gene length category, the highest-yielding mutant was selected and compared relative to this standard. At the same time, the enhancement of qPCR efficiency by Ton1914 and its mutants was verified. The experiment was conducted in a solution containing 5 μL of TB Green Premix, 1 μL of multiple template genes, 0.4 μL of an upstream primer, 0.4 μL of a downstream primer and 3.2 μL of Ton1914 and its mutants in liquid form, with the total reaction system comprising 10 μL. Then, qPCR was performed. The template genes used were randomly selected from existing target genes of various lengths in our laboratory.

### 2.10. UDP-Gal Synthesis

The previously established three-enzyme cascade synthesis system for UDP-Gal was used to determine the activity of the mutants [24]. The reaction was performed in a 1 mL solution containing 50 mM Na_2_HPO_4_-NaOH (pH 9.0), 20 mM Mg^2+^, 20 mM UTP, 20 mM Gal, 20 mM ATP, 0.1 mg Tth0595, 1 mg Tte0732 and different quantities of Ton1914 and its mutants. The generation of UDP-Gal was verified using high-performance liquid chromatography (HPLC), with the instrument connected to a 254 nm UV detector. The linear HPLC gradient on the Supersil AQ-C18 HPLC column consisted of solution A, comprising 40 mM triethylamine (adjusted with acetic acid to pH 6.0), and solvent B, based on solution A with the addition of 2% acetonitrile. After a 10 μL injection of each sample, solvent B was held at 0% for 5 min, increased from 0% to 100% in 5 min, held at 100% for 5 min and then reduced to 0% in 5 min.

## 3. Results

### 3.1. Selection of Mutation Sites

**Figure 1:**
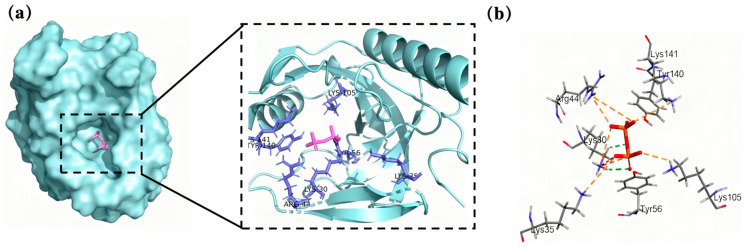
Molecular docking results. (**a**) Docking results for Ton1914 with a pyrophosphate molecule and (**b**) the interaction between them (the protein is blue and the pyrophosphate is purple).

In order to determine the binding site of the substrate molecule to PPase, molecular docking was performed using CDOCKER. Then, the optimal docking result was selected based on the molecular orientation of the substrate and the active site. Ligand and receptor structures were defined in Discovery Studio, using the Ligand Interaction program to identify amino acid residues and chemical bonds involved in the interactions between proteins and ligands (Figure 1). Pyrophosphate can form intermolecular hydrogen bonds with Lys30, an amino acid residue of Ton1914; furthermore, it electrostatically interacts with the amino acid residues Lys30, Lys35, Arg44, Tyr56, Lys105, Tyr140 and Lys141. Therefore, the subsequent selection of mutation sites focused on these amino acid residues and their nearby sites.

**Figure 2:**
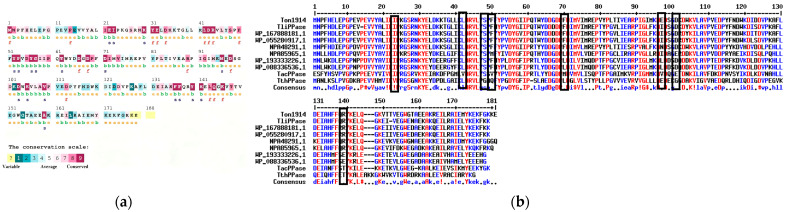
Ton1914 sequence analysis. (**a**) Ton1914 conservation analysis: e—amino acid residues exposed on the protein surface; b—amino acid residues buried within the protein structure; f—predicted functional amino acid residues; s—predicted structural amino acid residues. (**b**) A comparison of the amino acid sequence of Ton1914 with those of inorganic pyrophosphorylases derived from other thermophilic bacteria and inorganic pyrophosphorylases within the same family with a homology of 60–80%, with selected mutation sites in black boxes. In the sequence alignment results, amino acids with low conservation are represented by black letters, those with high conservation by blue letters, and completely conserved amino acids by red letters.

Consurf was used to perform sequence conservation analysis on Ton1914 and conduct a preliminary site screening based on this analysis (Figure 2a). In Figure 2, the amino acids marked in purple are fully conserved residues, which play crucial roles in the catalytic center and stable structure of the protein. Amino acids marked in blue are non-conserved and serve as potential sites for selection. On this basis, amino acid residues with non-conserved side chains could be screened out, narrowing the scope of modification. At the same time, the amino acid sequence of Ton1914 was compared with those of inorganic pyrophosphorylases derived from other thermophilic bacteria and inorganic pyrophosphorylases within the same family with homology values between 60 and 80% (Figure 2b). Based on the results of conservation analysis and multiple sequence alignment as well as HotSpot Wizard calculations, eight amino acid sites were ultimately selected for mutation.

### 3.2. Expression and Purification of Enzymes and Determination of Enzyme Activity

**Figure 3:**
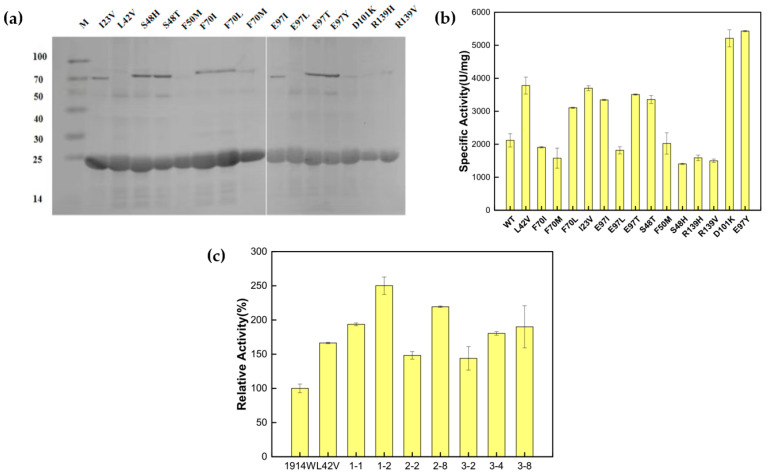
Results of construction, expression and enzyme activity determination of Ton1914 and its single mutants: (**a**) Expression of single mutants. Protein expression levels (mg/mL), left to right: 0.613, 0.593, 0.67, 0.476, 0.494, 0.724, 0.683, 0.592, 0.638, 0.611, 0.607, 0.587, 0.672, 0.358, 0.545; (**b**) enzyme activity of single mutants; (**c**) saturation mutation results for mutant L42V, in which mutations numbered 1-1, 1-2 and 2-8 were confirmed by sequencing to be L42W, L42F and L42C, respectively. (All assays were performed in triplicate, and the standard deviations of the biological replicates are represented by error bars).

After the mutation sites were determined and primers were designed (Table S1), the mutants were constructed using whole-plasmid PCR. The expression and purification of all mutants were induced (Figure 3a), and the enzyme activity of each mutant was measured (Figure 3b). The enzyme activity of the wild-type enzyme Ton1914 was 2100 U/mg, while of the mutants, E97Y demonstrated the highest enzyme activity at about 5400 U/mg, 2.57-fold that of the wild-type enzyme. The specific activity of D101K was about 5200 U/mg, 2.47-fold that of the wild-type enzyme. The enzyme activity of the other superior mutants also increased about 1.7-fold relative to the wild-type enzyme. According to these results, the three mutants with the greatest enzyme activity improvement, L42V, E97Y and D101K, were selected for saturation mutation. Based on the saturated mutation sites, thirteen pairs of degenerate primers (Table S2) were designed, and pET28a-Ton1914 was used as a template to perform full-plasmid PCR to achieve saturated mutations at the three sites. After a sufficient number of monoclonal colonies were selected and induced, the enzyme activity of their mutants was measured. The saturated mutations in E97Y and D101K did not produce other superior mutants (Figure S1), but compared to L42V, the mutants L42W, L42F and L42C showed higher enzyme activities. The enzyme activity of L42F was 2.15-fold that of the wild-type enzyme (Figure 3c).

**Figure 4:**
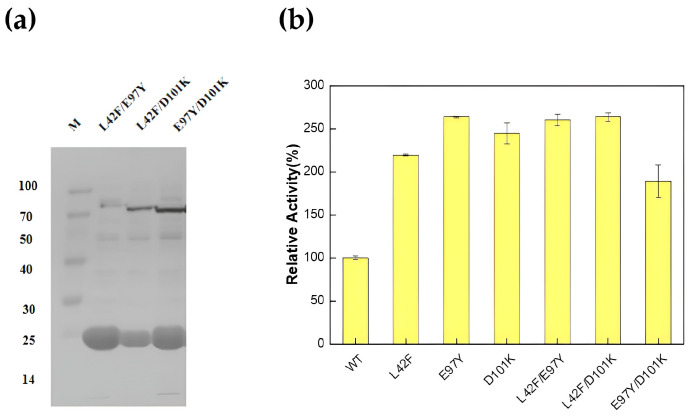
Results of construction, expression and enzyme activity determination of double mutants. (**a**) Expression and purification of double mutants. The expression levels of the three double mutants are as follows: L42F/E97Y, 0.623 mg/mL; L42F/D101K, 0.408 mg/mL; and E97Y/D101K, 0.678 mg/mL; (**b**) Enzyme activity comparison between double mutants and dominant single mutants. (All assays were performed in triplicate; error bars represent the standard deviations of biological replicates.).

According to the saturation mutation results, three double mutants, L42F/E97Y, L42F/D101K and E97Y/D101K, were chosen to further explore their enzyme activity. After their successful construction and expression (Figure 4a), the L42F/E97Y and L42F/D101K superimposed mutants still maintained high enzyme activity, 2.60- and 2.63-fold that of the wild-type enzyme, respectively. However, the mutant E97Y/D101K, a combination of the two most superior mutants, E97Y and D101K, did not exhibit considerable enzyme activity at 1.88-fold that of the wild-type enzyme (Figure 4b).

### 3.3. Enzymatic Properties of Ton1914 Mutants

**Figure 5:**
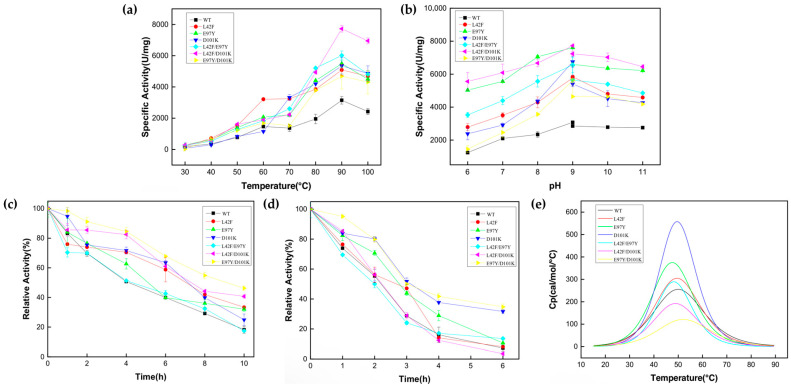
Enzymatic characterization of Ton1914 and its mutants. (**a**) Results of optimum reaction temperature determination; (**b**) results of optimal reaction pH determination; (**c**) stability of enzymes at 80 °C; (**d**) stability of enzymes at 90 °C; (**e**) results of thermal stability analysis. (All assays were performed in triplicate; error bars represent the standard deviations of biological replicates).

The optimum reaction temperature of Ton1914 and its mutants was determined (Figure 5a) to be 90 °C, at which the specific activity of L42F/D101K reached 7700 U/mg, about 2.5-fold that of the wild-type enzyme, while that of other mutants was about 1.5-fold that of Ton1914. Notably, the activities of the mutants were significantly enhanced at 50–70 °C compared to that of the wild-type enzyme, facilitating the use of Ton1914 at medium–high temperatures. The optimal reaction pH of Ton1914 was 9.0, with this value being consistent across mutants (Figure 5b). However, all mutants had higher enzyme activity than the wild-type enzyme at other pH values. At a pH of 6.0, the enzyme activity of L42F/D101K reached 5500 U/mg, 4.4-fold that of the wild-type enzyme, and it demonstrated high suitability in a wider range of pH values.

Since the optimal temperature of Ton1914 is quite high, the stability of its mutants was measured at 80 °C and 90 °C (Figure 5c,d). The half-life of Ton1914 at 80 °C was 4 h, and after 10 h, the remaining enzyme activity was 18% of the initial activity. For every mutant except L42F/D101K, thermal stability at 80 °C improved to varying degrees. Specifically, the half-life of E97Y/D101K at 80 °C reached 10 h. The results of a test conducted at 90 °C showed that the thermal stability of PPase further decreased. Compared to the wild-type enzyme, with its 2 h half-life, the E97Y/D101K mutant exhibited better thermal stability, with a half-life of approximately 3 h at 90 °C. The DSC thermal stability curve fitting results showed that the Tm value of the E97Y/D101K mutant increased by about 2 °C compared to the wild-type enzyme. This indicated that the E97Y/D101K had better thermal stability than the wild-type enzyme, which was consistent with the above results. Furthermore, the temperature stability results for pyrophosphatase revealed that the Tm value of the L42F/E97Y mutant was 2 °C lower than that of the wild-type enzyme, consistent with the finding that the former’s stability at high temperatures was not improved compared to the latter (Figure 5e).

### 3.4. Kinetic Analysis of Mutants

Using pyrophosphate as the substrate, the kinetic parameters of the inorganic pyrophosphatase Ton1914 and each mutant were determined (Table 1). In addition to the D101K mutation, the *K_m_* value of all mutants increased compared to the wild-type enzyme, indicating that the affinity of these mutants for substrates was reduced to varying degrees. At the same time, the *k_cat_* values of these mutants were significantly higher than those of the wild-type enzyme, meaning that with the exception of the D101K mutant, all mutants had favorable catalytic rate constants, with their *k_cat_*/*K_m_* values being significantly higher than those of the wild-type enzyme. The *k_cat_*/*K_m_* values of L42F and E97Y/D101K reached 1.84- and 1.50-fold that of the wild-type enzyme, indicating that the substrate conversion rate and enzyme catalytic rate constant of the mutants both increased. The increase in the catalytic rate constant compensates for the negative effect of the slight decrease in substrate affinity, with the overall result being a substantial increase in catalytic efficiency. The D101K’s *k_cat_*/*K_m_* value reached 4.22-fold that of the wild-type enzyme, and its affinity to the substrate was greatly enhanced, which was an important factor in its higher catalytic efficiency.

### 3.5. Structural Insights Based on Molecular Dynamics Simulation

The catalytic functions of Ton1914 could be divided into four regions according to its amino acid sequence. The two regions of amino acid residues 30–45 and 140–141 were mainly responsible for binding pyrophosphate and stabilizing the active pocket structure. The two regions of amino acid residues 66–71 and 97–103 were the main catalytic active centers, which bound with water molecules to act as nucleophiles to attack oxygen atoms and stabilized magnesium ions. Obviously, reducing the flexibility of the first two regions and enhancing the flexibility of the last two regions was more conducive to improving the enzyme activity of Ton1914.

**Figure 6:**
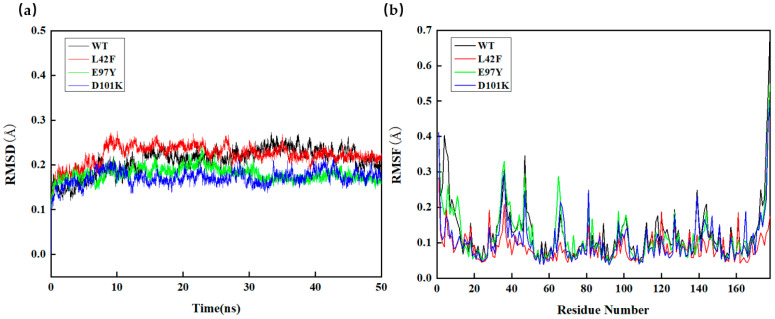
Molecular dynamics simulation of Ton1914 and mutants. (**a**) Root mean square deviation (RMSD) of wild-type enzyme and mutants; (**b**) root mean square fluctuation (RMSF) of wild-type enzyme and mutants.

The RMSD results of L42F, E97Y, and D101K were shown in Figure 6. The stability of the L42F showed no significant difference compared to the wild-type enzyme. The RMSD values of E97Y and D101K mutants were lower than that of the wild-type enzyme after 30 ns, indicating that they had more stable conformations. The RMSF value of L42F showed that its structure tended towards being more rigid, so the active pocket of L42F was more stable. L42F’s active site had stronger rigidity to facilitate substrate entry and exit, so its enzyme activity was higher than that of wild-type enzyme. For E97Y, its catalytic active center was more flexible and had a higher probability of contacting substrates and catalyzing reactions. Therefore, its enzyme activity was higher compared to the wild-type enzyme. The RMSF value of the D101K mutant did not indicate a substantial change in flexibility at that specific residue. However, a broader analysis revealed a noticeable reduction in RMSF values across a significant portion of the protein structure. Combined with the decreased global RMSD observed for this mutant, it was proposed that the D101K mutation enhanced the overall rigidity of the enzyme. This interpretation was further supported by the improved thermal stability of the D101K variant, consistent with the RMSD and large-scale RMSF trends. To facilitate clearer interpretation, the RMSD and RMSF results for all mutants have been provided in the Supplementary Materials (Figure S2).

### 3.6. Improving PCR Efficiency

**Figure 7:**
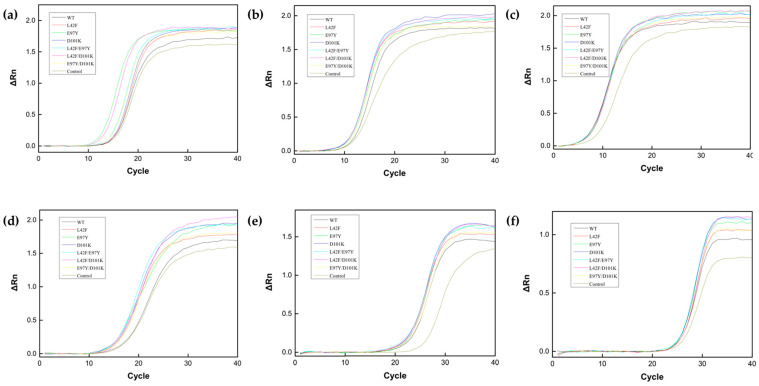
Results demonstrating the effect of Ton1914 and its mutants in enhancing qPCR. The lengths of the template genes are (**a**): 537 bp; (**b**): 1338 bp; (**c**): 2106 bp; (**d**): 2859 bp; (**e**): 5770 bp; (**f**): 8137 bp.

To validate PCR enhancement, six target genes of different lengths were used for qPCR, and the amount of inorganic pyrophosphatase added to the qPCR system was preliminarily optimized (Figure S3). It was found that Ton1914 at 0.7 μg/mL exhibited the best PCR enhancement and that an excessive quantity of PPase affected the PCR amplification efficiency. Therefore, in order to reduce the amount of inorganic pyrophosphatase in the PCR system and its impact on DNA polymerase to promote the reaction, it was necessary to improve the enzyme activity of Ton1914. The results showed that Ton1914 and its mutants reduced the number of qPCR cycles required to reach the threshold and improved the efficiency of gene amplification. Compared to the wild-type enzyme, four mutants, E97Y, D101K, L42F/E97Y and L42F/D101K, significantly enhanced the qPCR (Figure 7a–f).

**Figure 8:**
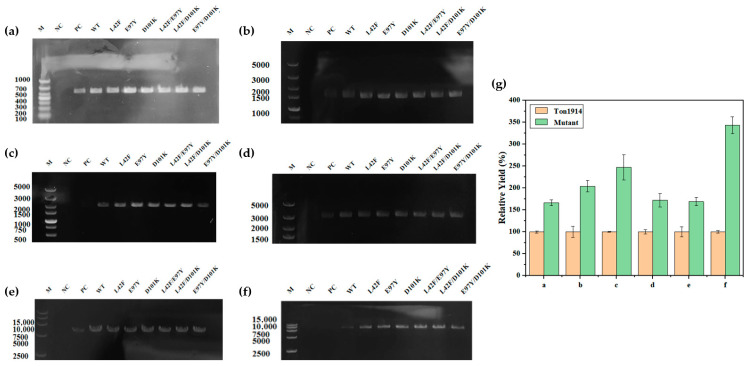
Results demonstrating the effect of Ton1914 and its mutants in enhancing PCR. The lengths of the template genes are (**a**): 537 bp; (**b**): 1338 bp; (**c**): 2106 bp; (**d**): 2859 bp; (**e**): 5770 bp; (**f**): 8137 bp; (**g**) Enhanced PCR efficiency of mutants compared to Ton1914. For the six template genes of different lengths, the mutants exhibiting the strongest improvement in PCR performance were: (**a**) L42F/E97Y; (**b**) E97Y/D101K; (**c**) D101K; (**d**) E97Y/D101K; (**e**) E97Y/D101K; (**f**) L42F/E97Y. Within each independent group, the yield of its own WT (wild-type enzyme) group was set as 100% for the comparison of relative yields. (All assays were performed in triplicate, and the standard deviations of the biological replicates are represented by error bars).

More intuitive PCR enhancement results were displayed through agarose gel electrophoresis, with the differences in product brightness demonstrating each mutant’s ability to promote the PCR. Evidently, for the control group without PPase, the amount of product after 20 cycles was minimal, and the target gene could be obtained, even in the reaction system in which this gene was excessively long (8137 bp). The addition of Ton1914 alleviated this situation but resulted in a lighter brightness of the target band, while higher brightness than the wild-type enzyme was observed in the experimental groups corresponding to the mutants, indicating that the Ton1914 mutants had a stronger PCR-enhancing effect (Figure 8a–g). When a long template gene (f) was used, the yield in the group supplemented with the mutant enzyme reached up to 3.4 times that of the group supplemented with Ton1914. This result clearly demonstrated that the mutant enzyme offered significantly greater potential for practical applications than Ton1914.

### 3.7. Improving UDP-Gal Synthesis Yield

**Figure 9:**
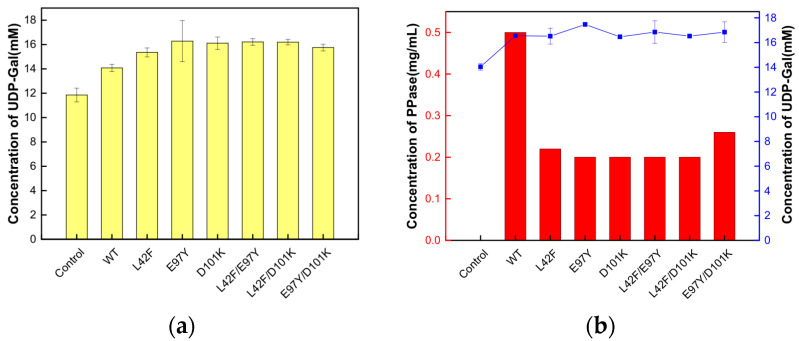
The influence of the addition of Ton1914 and its mutants on the yield of UDP-Gal. (**a**) The addition of 0.5 mg/mL of Ton1914 and its mutants in the cascade reaction of UDP-Gal synthesis; (**b**) The addition of 1000 U of Ton1914 and its mutants in the cascade reaction of UDP-Gal synthesis. (All assays were performed in triplicate, and the standard deviations of the biological replicates are represented by error bars).

In the salvage synthesis pathway of UDP-Gal, the presence of PPase made the reversible reaction catalyzed by UDP-glucose pyrophosphorylase tend toward UDP-Gal synthesis, improving the yield of UDP-Gal. In the existing UDP-Gal synthesis system, the amount of Ton1914 added reached 1 mg/mL because the synthesis conditions were not completely aligned with the optimal conditions for Ton1914. Increasing the enzyme activity of Ton1914 reduced the amount of PPase added to the system, which could increase cost-effectiveness and further promote the reaction. Overall, the enhanced activity of the mutants made them more effective in improving UDP-Gal synthesis (Figure 9a).

The same quantity of Ton1914 and each mutant (0.5 mg/mL) was added to the UDP-Gal synthesis system, and the reaction was terminated after 2 h. The UDP-Gal yield was 14.07 mM, representing an increase of 70.39%, after the addition of Ton1914. The mutants further increased the yield, with the most notable effect achieved by E97Y, which increased it by 4.42 mM, or 22.09%, compared to the control group. The results showed that each mutant could achieve the same yield of UDP-Gal as the wild-type enzyme with a lower enzyme amount, providing more-cost-effective and better-quality PPases for the synthesis of UDP-Gal (Figure 9b).

## 4. Discussions

Inorganic pyrophosphatase has been widely and effectively used to reduce the inhibition of pyrophosphate. However, in the face of certain harsh high-temperature reaction environments, such as those encountered in the PCR and high-temperature glycosylation reaction, only thermophilic inorganic pyrophosphatase can perform efficiently. Li provided an insight into the use of the thermophile PPase Ton1914 as a PCR enhancer and successfully verified this hypothesis [18]. In this study, the amount of Ton1914 added was optimized, and it was found that excessive PPase addition burdens the PCR system and may have counterproductive effects. Therefore, mutants with higher enzyme activity and stability may be better choices. PPase is also an important player in NDP-sugar synthesis and downstream glycosylation at high temperatures. Fischoder showed that PPase is generally indispensable in the synthesis of nucleotide sugars such as UDP-Gal and UDP-GalNAc [27]. Sun used hyperthermophile PPase to decompose pyrophosphoric acid to enhance the synthesis of glycosides in a one-pot synthesis system for nucleoside disaccharides. After incubation at 100 °C for 4 h, the activity of PPase remaining in this system was 100% [28]. However, many published experimental results have demonstrated that it is necessary to add a large quantity of PPase to the reaction system to yield a sufficient effect, which increases the reaction cost and is not conducive to the occurrence of the main reaction. Therefore, it is very important to modify PPase molecules by means of mutation to significantly improve their activity and stability. In preliminary research, Ton1914 has been confirmed to be a robust PPase that can be used in industrial catalysis. Nonetheless, there is still very high demand for enhancing the activity of Ton1914.

This study carried out the site-directed mutation of Ton1914 to improve its enzyme activity, reduce the amount required in practical applications and expand its scope of application. Ultimately, three single mutants and three double mutants were successfully constructed. The construction of the mutants did not cause excessive harm to the enzyme’s expression level. Except for L42F/D101K (0.408 mg/mL), the expression levels of the other five mutants were slightly higher than that of Ton1914 (0.58 mg/mL). Certainly, it was well-established that protein expression levels were intrinsically linked to both the specific strain utilized and the physiological growth state of the cells in each individual batch culture. It was found that all mutants induced varying degrees of improvement in enzyme activity and stability. Among them, L42F/D101K’s enzyme activity reached 7700 U/mg, 2.5-fold that of the wild-type enzyme under the optimal conditions. Moreover, in thermal stability tests, it was found that the incubation of E97Y/D101K at 80 °C resulted in a half-life of up to 10 h, which is much higher than the 4 h half-life of the wild-type enzyme. Since the enzyme activity of the double mutant E97Y/D101K decreased significantly compared to the two single mutants E97Y and D101K, L42F was not superimposed on E97Y/D101K to construct the triple mutant L42F/E97Y/D101K. As a result, the catalytic scenarios that Ton1914 mutants can cope with have become more diverse. Faced with various requirements, such as higher enzyme activity, better stability and the ability to persist in neutral or weakly alkaline catalytic environments, the mutants described in this work represent promising alternatives to Ton1914.

In terms of PCR enhancement, due to the improvement in enzyme activity, the amount of enzyme that needed to be added to the PCR system was greatly reduced, while the ability of mutants to reduce the inhibition of excessive foreign substances in the PCR was considerably higher than that of the wild-type enzyme. Each mutant was added to the reaction system for the one-pot synthesis of UDP-Gal, with the amount of enzyme kept constant, confirming that they all had better promotion effects than the wild-type enzyme. At the same time, the increase in enzyme activity allowed for a significant reduction in the amount of mutant added. The overall results indicate that this study provided more-cost-effective and superior-performance inorganic pyrophosphatases for high-demand PCRs and large-scale UDP-Gal synthesis in industry.

The high amount of PPase added to the one-pot reaction system is an important issue impacting the effects of other reactions and the purification of the product, with less PPase entailing reductions in the cost of the reaction and the impact of PPase on other reactions. It is clear that this study has yielded remarkable results on both fronts. For a long time, there have been few reports on the modification of inorganic pyrophosphatase by means of site-directed mutagenesis. Satho [29] investigated the role of two tryptophan residues in site-directed mutagenesis, suggesting that they may be responsible for the structural integrity and thermal stability of TthPPase. Shinoda [30] suggested that Val75 may be located at the interface between monomers in PPase derived from *Bacillus steatophilus* and that its hydrophobic interaction with the surrounding environment may play a key role in the thermal stability of the enzyme and oligomeric subunit interactions. In fact, most previous studies, including those mentioned above, explored key amino acid residues of PPase, and there are few reports on the activity and thermal stability of PPase. This study has expanded the body of research on PPase molecular evolution and has demonstrated the status of site-directed mutagenesis as a powerful method for PPase molecular modification.

## 5. Conclusions

In order to improve the activity of the thermophilic inorganic pyrophosphatase Ton1914, mutation sites were screened through molecular docking and sequence analysis and alignment. Then, through site-directed, saturation and superimposed mutations, six superior mutants were obtained. These mutants exhibit improvements in terms of enzyme activity and thermal stability to varying degrees. It was found that the enhancement effect on PCR was more evident when the mutants were used. Compared to the wild-type enzyme, the groups with mutants obtained more PCR products, and the longer the gene template, the more evident the effect of enhancing the PCR. Moreover, in the catalytic system within the UDP-Gal synthesis reaction, the mutants were proven to be better candidates. This study expands the literature on the molecular evolution of the PPase family. Specifically, PPases with better properties were obtained using site-directed mutagenesis, which is a fast and accurate molecular modification method. This paper thus demonstrates that mutants of Ton 1914 can be developed into stable and efficient PCR enhancers. Valuable future research directions in this regard include the pilot scale-up and industrial application of Ton1914 mutants in nucleotide–glycoside synthesis or other biocatalytic systems.

## Figures and Tables

**Table 1:** The kinetic parameters of Ton1914 and its mutants.

Enzymes	*K_m_* (mM)	*k_cat_* (s^−1^)	*k_cat_*/*K_m_* (mM^−1^·s^−1^)
Ton1914	3.03 ± 0.18 × 10^−4^	7.38 ± 0.20 × 10^3^	2.11 × 10^7^
L42F	3.18 ± 0.05 × 10^−4^	1.24 ± 0.17 × 10^4^	3.88 × 10^7^
E97Y	5.94 ± 0.14 × 10^−4^	1.71 ± 0.08 × 10^4^	2.87 × 10^7^
D101K	1.03 ± 0.04 × 10^−4^	9.13 ± 0.06 × 10^3^	8.89 × 10^7^
L42F/E97Y	8.49 ± 0.30 × 10^−4^	2.00 ± 0.13 × 10^4^	2.36 × 10^7^
L42F/D101K	6.34 ± 0.82 × 10^−4^	1.68 ± 0.08 × 10^4^	2.65 × 10^7^
E97Y/D101K	3.48 ± 0.27 × 10^−4^	1.10 ± 0.26 × 10^4^	3.17 × 10^7^

All assays were performed in triplicate.

## Data Availability

Data are contained within the article and Supplementary Materials.

## Supplementary Materials

The following supporting information can be downloaded at: https://www.mdpi.com/article/10.3390/biom15101395/s1, Table S1: Primers for single mutants; Table S2: Sequences of degenerate primers used for saturation mutations; Figure S1: The saturated mutation results against E97Y and D101K; Figure S2: The RMSD and RMSF plots for all single mutants; Figure S3: Optimization of PPase addition in qPCR.
