# Supplementary material for: Enhancement of Activity of Thermophilic Inorganic Pyrophosphatase Ton1914 via Site-Directed Mutagenesis
Source: Biomolecules. 2025 Sep 30;15(10):1395. doi: 10.3390/biom15101395 (PMC12562645; doi:10.3390/biom15101395)

Supplementary Materials

Table S1. Primers for single mutants.

Primers	Sequences (5′-3′)
I23V-UP	TATAGAGGTTCCGAAGGGAAGCAGGAACA
I23V-DOWN	TTCGGAACCTCTATAAGAGCGTAAACGACCTCT
L42V-UP	TCTCAAGGTCGATAGAGTGCTCTACAGCCCG
L42V-DOWN	CTATCGACCTTGAGAAGGCCGGTCTTC
S48H-UP	GCTCTACCATCCGTTCTTCTACCC
S48H-DOWN	AACGGATGGTAGAGCACTCTATCAA
S48T-UP	GCTCTACACGCCGTTCTTCTACCC
S48T-DOWN	AACGGCGTGTAGAGCACTCTATCAA
F50M-UP	AGTGCTCTACAGCCCGATGTTCTAC
F50M-DOWN	CAACCGGGTAGAACATCGGGCTG
F70I-UP	CGACCCCATCGACATAATGGTCATCATGCGCGA
F70I-DOWN	ATGTCGATGGGGTCGCCGTCGTCGTA
F70L-UP	CGACCCCTTAGACATAATGGTCATCATGCGCGA
F70L-DOWN	ATGTCTAAGGGGTCGCCGTCGTCGTA
F70M-UP	CGACCCCATGGACATAATGGTCATCATGCGCGA
F70M-DOWN	ATGTCCATGGGGTCGCCGTCGTCGTA
E97I-UP	GAAGATGATCGACAGCGGAGACAAAGACTGG
E97I-DOWN	CTGTCGATCATCTTCATGATGCCTATCGGCC
E97L-UP	GAAGATGTTAGACAGCGGAGACAAAGACTGG
E97L-DOWN	CTGTCTAACATCTTCATGATGCCTATCGGCC
E97T-UP	GAAGATGACCGACAGCGGAGACAAAGACTGG
E97T-DOWN	CTGTCGGTCATCTTCATGATGCCTATCGGCC
E97Y-UP	GAAGATGTACGACAGCGGAGACAAAGACTGG
E97Y-DOWN	CTGTCGTACATCTTCATGATGCCTATCGGCC
D101K-UP	CAGCGGAAAGAAAGACTGGAAGGTTCTCGCCG
D101K-DOWN	TCTTTCTTTCCGCTGTCCTCCATCTTCATG
R139H-UP	GCCCACTTCTTCCAGCACTACAAGG
R139H-DOWM	TTTGCCCTGGAGCTGTGTGTACCTC
R139V-UP	TTCCAGGTATACAAGGAGCTCCAGG
P139V-DOWM	CCTTGTATACCTGGAAGAAGTGGGC

Table S2. Sequences of degenerate primers used for saturation mutations.

Number	Primers	Sequences (5′-3′)	Corresponding amino acids
1	L42-1	TCTCAAGVVCGATAGAGTGCTCTACAGCCCG	A, D, G, H, N, P, R, S, T
	L42-2	CTATCGNNCTTGAGAAGGCCGGTCTTC	
2	L42-3	TCTCAAGTDYGATAGAGTGCTCTACAGCCCG	C, F, Y
	L42-4	CTATCRVACTTGAGAAGGCCGGTCTTC	
3	L42-5	TCTCAAGVWAGATAGAGTGCTCTACAGCCCG	E, I, K, Q
	L42-6	CTATCTWNCTTGAGAAGGCCGGTCTTC	
4	L42W-1	TCTCAAGTGGGATAGAGTGCTCTACAGCCCG	W
	L42W-2	CTATCCCACTTGAGAAGGCCGGTCTTC	
5	E97-1	GAAGATGVVCGACAGCGGAGACAAAGACTGG	A, D, G, H, N, P, R, S, T
	E97-2	CTGTCGBBCATCTTCATGATGCCTATCGGCC	
6	E97-3	GAAGATGNTTGACAGCGGAGACAAAGACTGG	F, I, L, V
	E97-4	CTGTCAANCATCTTCATGATGCCTATCGGCC	
7	E97-5	GAAGATGTGBGACAGCGGAGACAAAGACTGG	C, W
	E97-6	CTGTCVCACATCTTCATGATGCCTATCGGCC	

8	E97-7	GAAGATGMAAGACAGCGGAGACAAAGACTGG	K, Q
	E97-8	CTGTCTTKCATCTTCATGATGCCTATCGGCC	
9	D101Q-1	CAGCGGACAAAAAGACTGGAAGGTTCTCGCCG	Q
	D101Q-2	TCTTTTTGTCCGCTGTCCTCCATCTTCATG	
10	D101W-1	CAGCGGATGGAAAGACTGGAAGGTTCTCGCCG	W
	D101W-2	TCTTTCCATCCGCTGTCCTCCATCTTCATG	
11	D101-1	CAGCGGANSTAAAGACTGGAAGGTTCTCGCCG	A, C, G, P, R, S, T
	D101-2	TCTTTASNTCCGCTGTCCTCCATCTTCATG	
12	D101-3	CAGCGGANTTAAAGACTGGAAGGTTCTCGCCG	F, I, L, V
	D101-4	TCTTTAANTCCGCTGTCCTCCATCTTCATG	
13	D101-5	CAGCGGANACAAAGACTGGAAGGTTCTCGCCG	E, H, N, P
	D101-6	TCTTTGTNTCCGCTGTCCTCCATCTTCATG	

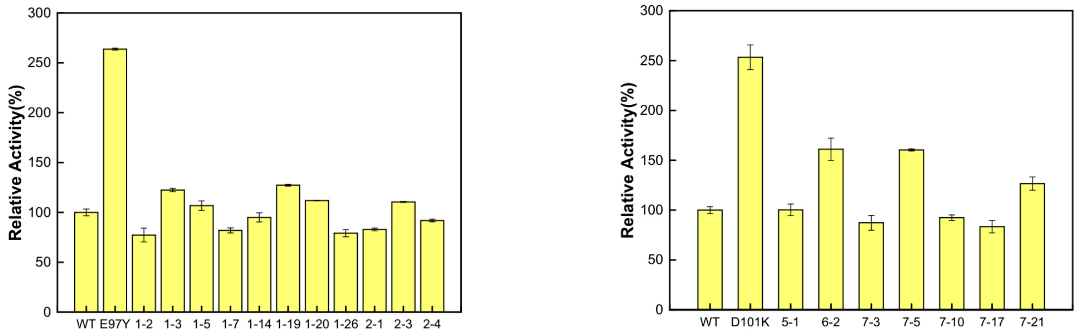

(a) (b)

Figure S1. The saturated mutation results against **(a)** E97Y and **(b)**D101K.

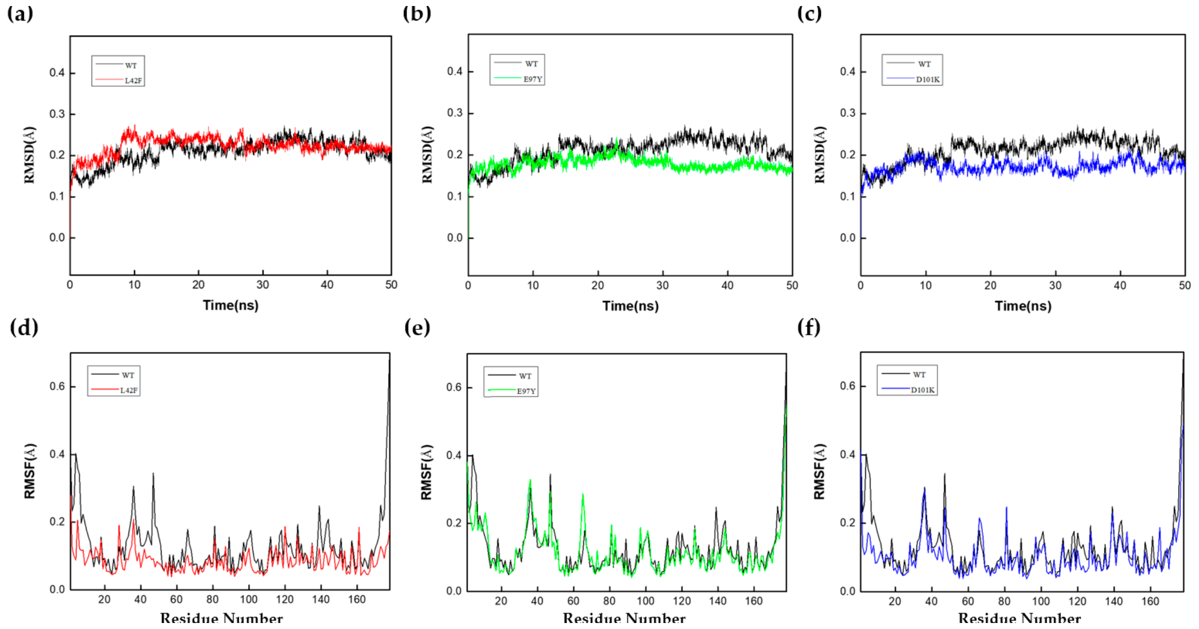

Figure S2. The RMSD and RMSF plots for all single mutants. In panels **(a–f)**, each plot was clearly labeled to indicate which specific mutant it corresponded to.

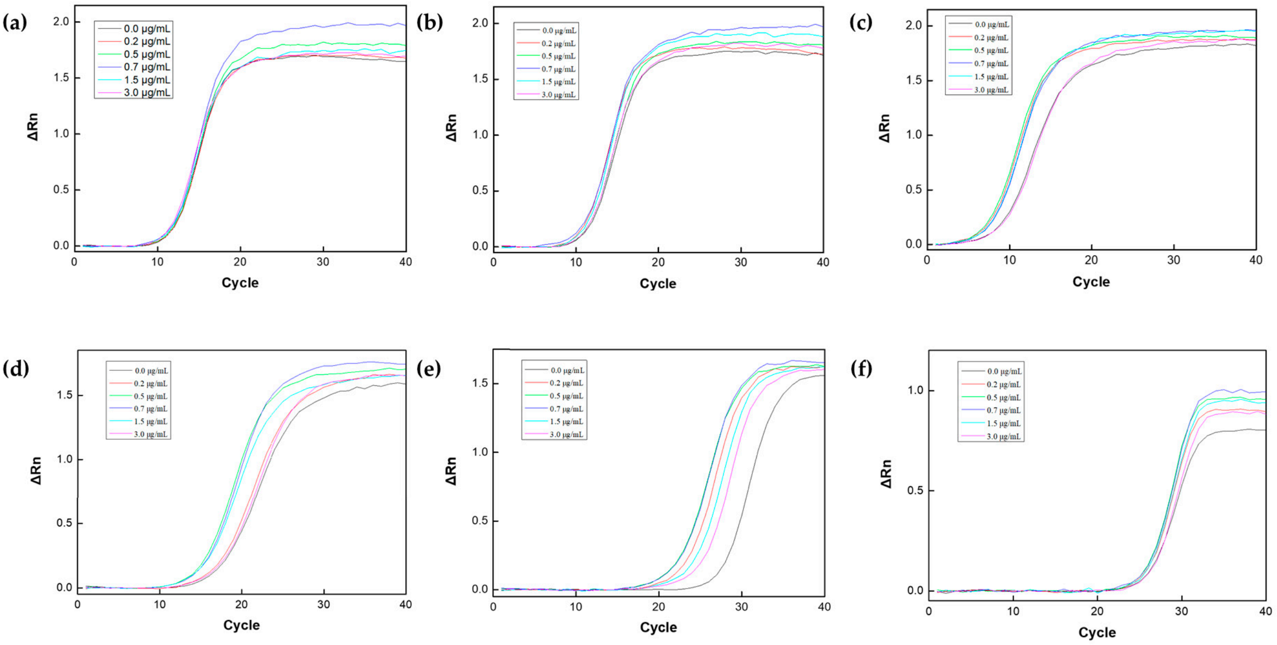

Figure S3. Optimization of PPase addition in qPCR reaction: The amount of PPase added was set to 0-3 μg/mL, and different amounts of PPase were added to the qPCR system of different gene lengths to optimize the amount of PPase. The lengths of each template gene are:**(a)**: 537bp; **(b)**: 1338bp; **(c)**: 2106bp; **(d)**: 2859bp; **(e)**: 5770bp; **(f)**: 8137bp.The amount of PPase added at 0.7 μg/mL was selected.

Rationale for Selecting the Initial Eight Mutation Sites:

Isoleucine 23 (I23): Position 23 is located in close proximity to Lys30, a residue that forms both hydrogen and ionic bonds with the substrate. Based on sequence alignment results, we constructed the mutant I23V.

Leucine 42 (L42): Interaction analysis revealed that Arg44 participates in the catalytic process by interacting with the substrate. Additionally, position 43 is highly conserved. Considering the sequence alignment, we decided to mutate Leu42 and constructed the mutant L42V.

Serine 48 (S48): This site was selected using the HotSpot Wizard tool, which integrates structural, functional, and evolutionary data from multiple databases to identify "hotspots"—non-conserved residues near the active site or within substrate access channels—for protein engineering. Based on evolutionary analysis, the tool recommended mutations to histidine or threonine. Thus, we constructed mutants S48H and S48T.

Phenylalanine 50 (F50): Supported by recommendations from HotSpot Wizard and sequence alignment showing variability at this position, we constructed the mutant F50M.

Phenylalanine 70 (F70): The catalytic center of inorganic pyrophosphatase involves residues Asp66, Asp68, and Asp71. We focused on the non-conserved position 70 with the aim of reducing side chain steric hindrance near the catalytic center. Based on sequence alignment, we constructed mutants F70I, F70L, and F70M.

Glutamate 97 (E97): This residue is adjacent to the catalytic core residues Asp98 and Asp103, both of which strongly interact with metal ions involved in catalysis. Sequence alignment revealed that position 97 is not strictly conserved and can be occupied by non-polar residues. Therefore, we

constructed mutants E97I and E97L. Additionally, to explore the effect of non-ionizable polar side chains, we also generated mutants E97T and E97Y.

Aspartate 101 (D101): Similar to position 97, this site was selected due to its functional context. However, sequence alignment showed that it is typically occupied by either aspartate or glutamate. To investigate the effect of altering the local charge environment without disrupting the protein structure, we performed computational analysis using FireProt (https://loschmidt.chemi.muni.cz/fireprotweb/). The results suggested that mutating this site to lysine could enhance thermal stability, leading to the construction of mutant D101K.

Arginine 139 (R139): This residue is situated near Tyr140 and Lys141. Combined with hotspot predictions from HotSpot Wizard, we constructed mutants R139H and R139V.

The raw data for the determination of kinetic parameters:

All raw data related to the kinetic parameters were provided here. All data points were included within the curve, and their associated error bars were displayed. The error bars for most data points were too small to be visible. All the final calculated values of K_m and k_{cat} were summarized in the table below. All assays were performed in triplicate, and the standard deviations of the biological replicates are represented by error bars and purpose bands are indicated by arrows.

Ton1914-WT										
[S]mol/L	0.00005	0.0001	0.00015	0.0002	0.00025	0.0003	0.0004	0.0006	0.0008	0.001
Group1	0.237	0.369	0.498	0.682	0.76	0.909	0.961	0.989	0.968	0.906
Group2	0.232	0.394	0.527	0.659	0.794	0.921	0.966	0.963	0.991	1.081
Group3	0.239	0.374	0.511	0.672	0.788	0.914	0.959	0.971	0.978	0.92
V(mol/L·s）										
Group1	1.11519E-06	1.9656E-06	2.79668E-06	3.98209E-06	4.4846E-06	5.44453E-06	5.77954E-06	5.95993E-06	5.82464E-06	5.4252E-06
Group2	1.08298E-06	2.12666E-06	2.98351E-06	3.83391E-06	4.70365E-06	5.52184E-06	5.81175E-06	5.79242E-06	5.97281E-06	6.55263E-06
Group3	1.12808E-06	1.99781E-06	2.88043E-06	3.91767E-06	4.66499E-06	5.47674E-06	5.76665E-06	5.84396E-06	5.88906E-06	5.5154E-06
L42F										
[S]mol/L	0.00005	0.0001	0.00015	0.0002	0.00025	0.0003	0.0004	0.0006	0.0008	0.001
Group1	0.191	0.386	0.539	0.68	0.793	0.9	0.956	0.959	0.965	0.938
Group2	0.233	0.393	0.545	0.722	0.84	0.918	1.014	1.115	1.022	0.985
Group3	0.212	0.3895	0.542	0.701	0.8165	0.909	0.985	1.037	0.9935	0.9615
V(mol/L·s）										
Group1	8.18838E-07	2.07512E-06	3.06082E-06	3.9692E-06	4.6972E-06	5.38655E-06	5.74733E-06	5.76665E-06	5.80531E-06	5.63136E-06
Group2	1.08942E-06	2.12022E-06	3.09947E-06	4.23979E-06	0.000005	5.50251E-06	6.12099E-06	6.77168E-06	6.17253E-06	5.93416E-06
Group3	9.5413E-07	2.09767E-06	3.08014E-06	4.1045E-06	4.8486E-06	5.44453E-06	5.93416E-06	6.26917E-06	5.98892E-06	5.78276E-06
E97Y										
[S]mol/L	0.00005	0.0001	0.00015	0.0002	0.00025	0.0003	0.0004	0.0006	0.0008	0.001
Group1	0.225	0.369	0.521	0.69	0.78	1.005	1.271	1.509	1.427	1.547
Group2	0.227	0.36	0.536	0.784	0.797	1.01	1.253	1.424	1.516	1.532
Group3	0.231	0.382	0.544	0.74	0.801	1.02	1.268	1.503	1.519	1.537
V(mol/L·s）										
Group1	1.03788E-06	1.9656E-06	2.94485E-06	4.03363E-06	4.61345E-06	6.06301E-06	7.7767E-06	9.31001E-06	8.78173E-06	9.55483E-06
Group2	1.05077E-06	1.90761E-06	3.04149E-06	4.63922E-06	4.72297E-06	6.09522E-06	7.66074E-06	8.7624E-06	9.35511E-06	9.45819E-06

Group3	1.07654E-06	2.04935E-06	3.09303E-06	4.35575E-06	4.74874E-06	6.15964E-06	7.75738E-06	9.27136E-06	9.37444E-06	9.4904E-06
D101K										
[S]mol/L	0.00005	0.0001	0.00015	0.00025	0.0003	0.0004	0.0006	0.0008	0.0001	
Group1	0.248	0.419	0.544	0.513	0.58	0.643	0.65	0.66	0.702	
Group2	0.243	0.422	0.552	0.567	0.565	0.674	0.677	0.696	0.713	
Group3	0.245	0.42	0.54	0.543	0.571	0.685	0.635	0.687	0.697	
V(mol/L·s）										
Group1	1.18606E-06	2.28772E-06	3.09303E-06	2.89331E-06	3.32496E-06	3.73083E-06	3.77593E-06	3.84036E-06	4.11094E-06	
Group2	1.15385E-06	2.30705E-06	3.14457E-06	3.24121E-06	3.22832E-06	3.93055E-06	3.94988E-06	4.07228E-06	4.18181E-06	
Group3	1.16673E-06	2.29416E-06	3.06726E-06	3.08659E-06	3.26698E-06	4.00142E-06	3.67929E-06	4.0143E-06	4.07873E-06	
L42F/E97Y										
[S]mol/L	0.00005	0.0001	0.00015	0.0002	0.00025	0.0003	0.0004	0.0006	0.0008	0.001
Group1	0.214	0.384	0.551	0.727	0.829	1.044	1.346	1.75	1.814	1.841
Group2	0.231	0.39	0.542	0.714	0.846	0.957	1.291	1.67	1.823	1.837
Group3	0.225	0.387	0.547	0.731	0.836	1.002	1.3	1.71	1.813	1.84
V(mol/L·s）										
Group1	9.67015E-07	2.06223E-06	3.13813E-06	4.272E-06	4.92913E-06	6.31426E-06	8.25989E-06	1.08626E-05	1.1275E-05	1.14489E-05
Group2	1.07654E-06	2.10089E-06	3.08014E-06	4.18825E-06	5.03865E-06	5.75377E-06	7.90555E-06	1.03472E-05	1.13329E-05	1.14231E-05
Group3	1.03788E-06	2.08156E-06	3.11236E-06	4.29777E-06	4.97423E-06	6.04368E-06	7.96354E-06	1.06049E-05	1.12685E-05	1.14425E-05
L42F/D101K										
[S]mol/L	0.00005	0.0001	0.00015	0.0002	0.00025	0.0003	0.0004	0.0006	0.0008	0.001
Group1	0.233	0.445	0.584	0.763	0.906	1.071	1.341	1.825	2.24	1.968
Group2	0.283	0.429	0.564	0.775	0.896	1.078	1.374	1.846	2.182	1.8
Group3	0.251	0.432	0.571	0.77	0.9	1.059	1.36	1.833	2.21	1.879
V(mol/L·s）										
Group1	1.08942E-06	2.45522E-06	3.35073E-06	4.50393E-06	5.4252E-06	6.48821E-06	8.22768E-06	1.13458E-05	1.40195E-05	1.22671E-05
Group2	1.41154E-06	2.35215E-06	3.22188E-06	4.58124E-06	5.36078E-06	6.53331E-06	8.44028E-06	1.14811E-05	1.36458E-05	1.11848E-05
Group3	1.20539E-06	2.37147E-06	3.26698E-06	4.54903E-06	5.38655E-06	6.4109E-06	8.35008E-06	1.13974E-05	1.38262E-05	1.16937E-05
E97Y/D101K										
[S]mol/L	0.00005	0.0001	0.00015	0.0002	0.00025	0.0003	0.0004	0.0006		
Group1	0.237	0.38	0.55	0.68	0.81	0.931	1.034	1.092		
Group2	0.214	0.367	0.569	0.694	0.776	0.947	1.094	1.014		
Group3	0.223	0.371	0.561	0.689	0.8	0.937	1.054	1.011		
V(mol/L·s）										
Group1	1.11519E-06	2.03646E-06	3.13168E-06	3.9692E-06	4.80673E-06	5.58626E-06	6.24984E-06	6.6235E-06		
Group2	9.67015E-07	1.95271E-06	3.25409E-06	4.0594E-06	4.58768E-06	5.68934E-06	6.63639E-06	6.12099E-06		
Group3	1.025E-06	1.97848E-06	3.20255E-06	4.02719E-06	4.7423E-06	5.62492E-06	6.37869E-06	6.10166E-06		

Ton1914-WT		Group 1	Group 2	Group 3	Average	Error	k_{cat} /K_m
	K_m	3.147×10^{-4}	3.113×10^{-4}	2.821×10^{-4}	3.03×10-4	1.79×10^{-5}	2.11×10^{7}
	k_{cat}	7497	7486	7148	7377	198.49	
L42F		Group 1	Group 2	Group 3	Average	Error	k_{cat} /Km
	K_m	3.328×10^{-4}	3.192×10^{-4}	3.13×10^{-4}	3.18×10^{-4}	4.9×10^{-6}	3.88×10^{7}
	k_{cat}	12501	12148	12359	12360	168.25	
E97Y		Group 1	Group 2	Group 3	Average	Error	k_{cat} /Km
	K_m	6.117×10^{-4}	5.902×10^{-4}	5.807×10^{-4}	5.94×10^{-4}	1.36×10^{-5}	2.87×10^{7}
	k_{cat}	17125	16978	17052	17050	82.31	
D101K		Group 1	Group 2	Group 3	Average	Error	k_{cat} /Km
	K_m	1.059×10^{-4}	1.025×10^{-4}	9.910×10^{-5}	1.03×10^{-4}	3.49×10^{-6}	8.89×10^{7}
	k_{cat}	9125	9195	9064	9128	62.13	
L42F/E97Y		Group 1	Group 2	Group 3	Average	Error	k_{cat} /Km
	K_m	8.189×10^{-4}	8.467×10^{-4}	8.812×10^{-4}	8.49×10^{-4}	3.02×10^{-5}	2.36×10^{7}
	k_{cat}	19824	20011	20167	20010	134.26	
L42F/D101K		Group 1	Group 2	Group 3	Average	Error	k_{cat} /Km
	K_m	7.421×10^{-4}	6.341×10^{-4}	5.563×10^{-4}	6.34×10^{-4}	8.23×10^{-5}	2.65×10^{7}
	k_{cat}	18250	16520	15570	16780	867.60	
E97Y/D101K		Group 1	Group 2	Group 3	Average	Error	k_{cat} /Km
	K_m	3.720×10^{-4}	3.269×10^{-4}	3.511×10^{-4}	3.48×10^{-4}	2.74×10^{-5}	3.17×10^{7}
	k_{cat}	11285	10710	11095	11030	264.21	

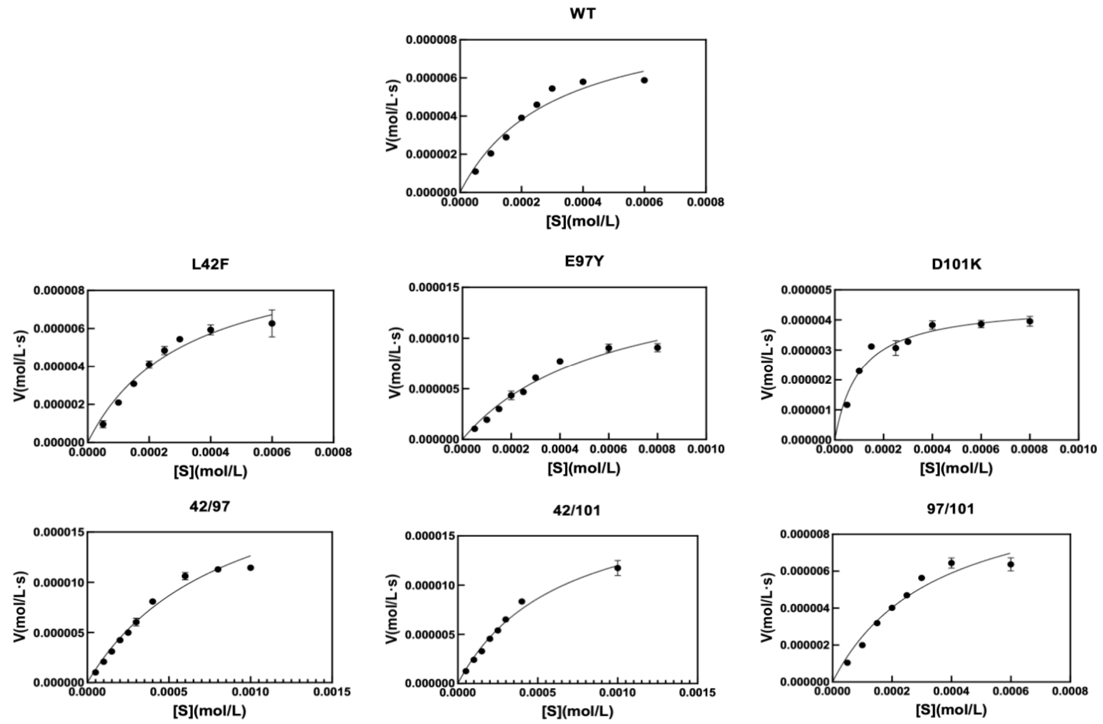

Supplement: Supplementary file 1 [file biomolecules-15-01395-s001.zip › biomolecules-3840876-supplementary.pdf]
