# Supplementary figures and images for: Enhancement of Activity of Thermophilic Inorganic Pyrophosphatase Ton1914 via Site-Directed Mutagenesis
Source: Biomolecules. 2025 Sep 30;15(10):1395. doi: 10.3390/biom15101395 (PMC12562645; doi:10.3390/biom15101395)

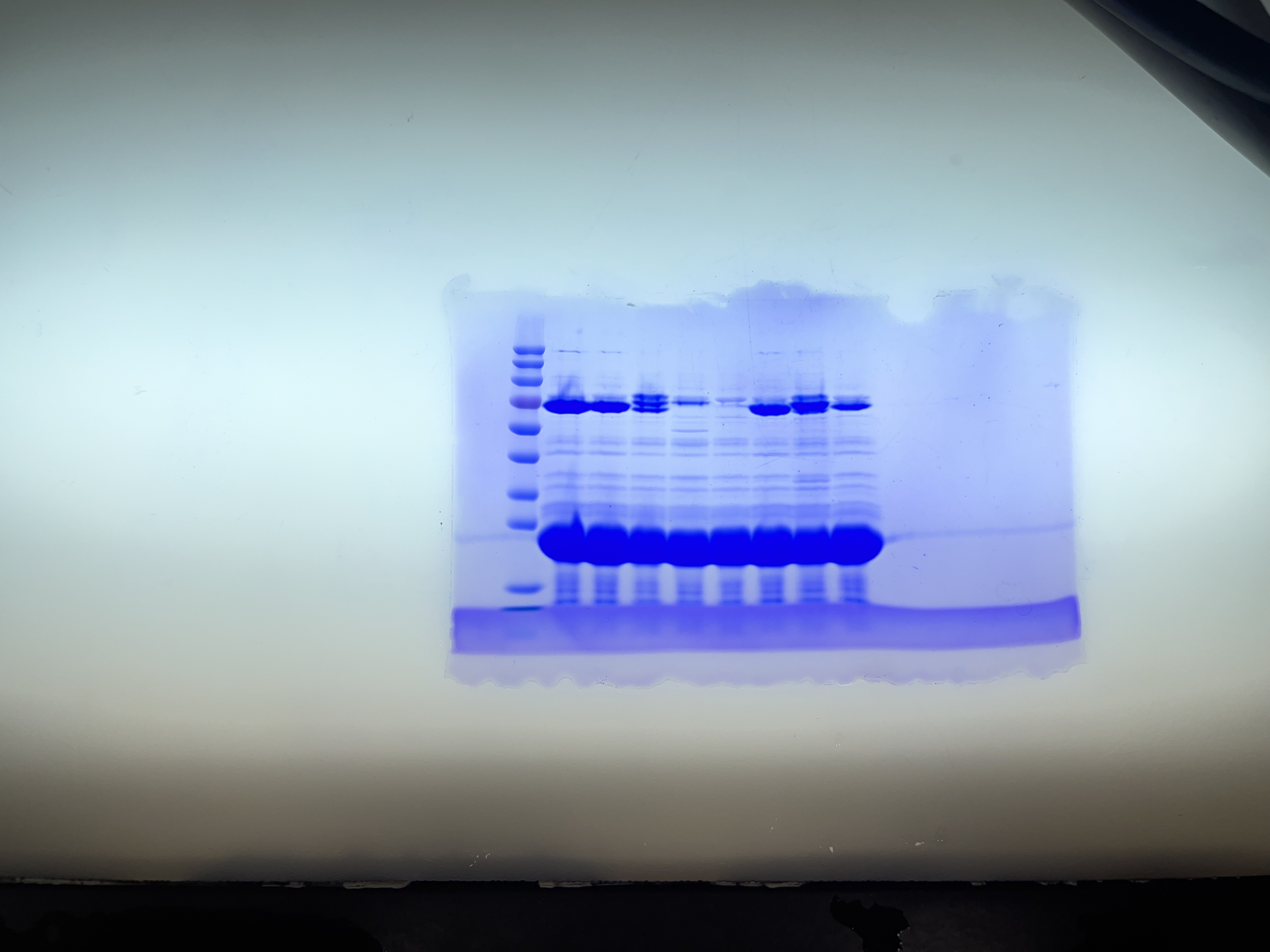

Supplement: Supplementary file 1 [file biomolecules-15-01395-s001.zip › Figure3-left.jpg]

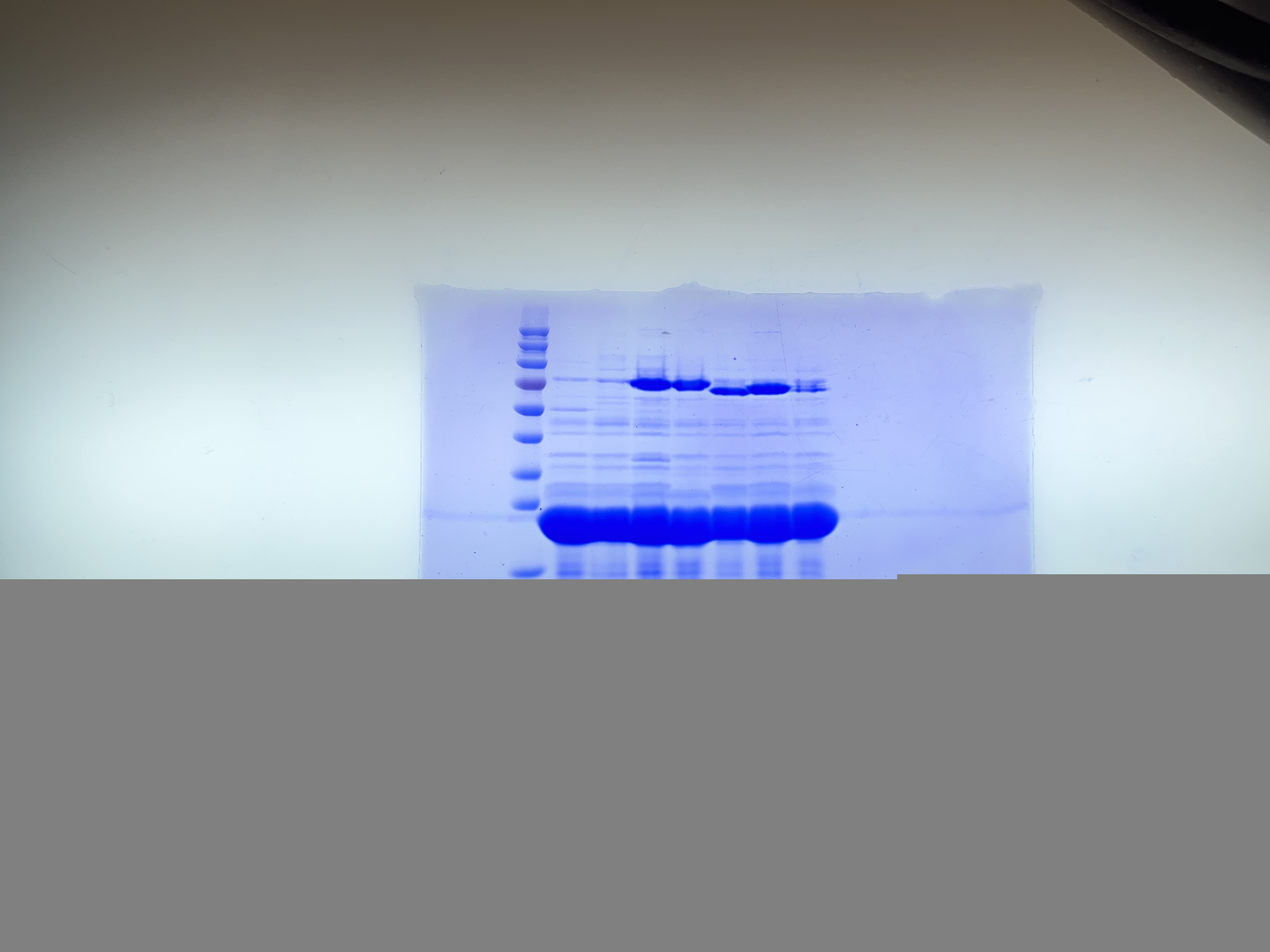

Supplement: Supplementary file 1 [file biomolecules-15-01395-s001.zip › Figure3-right.jpg]
